# Quality and content evaluation of websites with information about immune checkpoint inhibitors: An environmental scan

**DOI:** 10.1371/journal.pone.0275676

**Published:** 2022-10-10

**Authors:** Juan Ignacio Ruiz, Gagandeep Singh, McKenna Erck, Yimin Geng, Maria E. Suarez-Almazor, Maria A. Lopez-Olivo

**Affiliations:** 1 Department of Health Services Research, The University of Texas MD Anderson Cancer Center, Houston, TX, United States of America; 2 Division of Gastroenterology & Hepatology, Johns Hopkins University, Baltimore, MD, United States of America; 3 Texas A&M University, College Station, TX, United States of America; 4 Research Medical Library, The University of Texas MD Anderson Cancer Center, Houston, TX, United States of America; IRCCS Giovanni Paolo II Cancer Hospital, ITALY

## Abstract

**Background:**

Trustworthy educational information for patients is critical for increasing their knowledge base and preparing them for shared decision making with clinicians. As the internet has become an important source of health information for many patients, the purpose of this study was to assess the quality and content of websites with educational content about immune checkpoint inhibitors.

**Methods:**

We performed an environmental scan of the currently available websites providing educational information for patients about immune checkpoint inhibitors. We used three search engines: Google, Bing, and Yahoo! (9/20/2021). Two independent investigators selected relevant uniform resource locators (URLs), appraised the quality of the websites, and collected their characteristics. We evaluated the accuracy, completeness, technical elements, design and aesthetics, readability, usability, and accessibility of the websites. The user experience was also evaluated.

**Results:**

We identified 37 websites for analysis. In 10 websites (27%), it was not possible to know the source of the information provided. Thirty-three (89%) provided a definition with a simple explanation of cancer and treatment and 30 (81%) on complications of immune checkpoint inhibitors; only seven (19%) provided information about the balance between risks and benefits. Thirty-five (95%) provided a statement of purpose. Regarding the design, all 37 (100%) had appropriate visual aspects, typography, and grammar. Thirty-six (97%) were well organized. For most of the websites (n = 35, 95%) the content was easy to find. Only two websites had a readability score of 6, while the others had higher scores. Regarding the user experience, the overall quality of websites was rated as excellent in 16 (43%), good in 14 (38%), and fair in 7 (19%).

**Conclusions:**

Our findings reveal that websites with information about immune checkpoint inhibitors mostly have general information about cancer, the treatments, and adverse events. Few websites provide information about the balance between harms and benefits of treatment, costs, the source of the information, or the hierarchy of evidence. These findings identify the gap in the quality and content of websites for patients treated with immune checkpoint inhibitors and can help website creators and developers.

## Introduction

Immune checkpoint inhibitors (ICIs) are an increasingly used type of cancer immunotherapy that has been shown to improve the prognosis of patients with different types of cancers [1–5]. By blocking the function of key checkpoint factors, ICIs increase the activation of T cells, leading to a more vigorous response against tumors [6]. Monoclonal antibodies approved by the U.S. Food and Drug Administration (FDA) for cancer treatment include the programmed cell death 1 (PD-1) inhibitors nivolumab, pembrolizumab, cemiplimab, and dostarlimab and the programmed death ligand 1 (PD-L1) inhibitors atezolizumab, durvalumab, and avelumab [6]. Another “brake” on immune cells that negatively regulates activation of T-cells (CD4+ and CD8+) is cytotoxic T-lymphocyte-associated protein 4 (CTLA-4); the antibody ipilimumab inhibits CTLA-4 function and is also used to treat cancer. Recently, the FDA has approved relatlimab, a checkpoint inhibitor that targets lymphocyte-activation gene 3 (LAG-3), for the treatment of melanoma. Immune checkpoint inhibitors have transformed dramatically cancer management. Moreover, more patients have become eligible for ICI therapy over the years, including about 43.6% of patients with cancer in 2018 [7]. However, not all cases respond equally to ICI treatment, and different predictors of response have been suggested (e.g., PD-L1 expression, tumor-infiltrating lymphocytes, tumor mutational burden, tumor-associated macrophages) [8, 9].

Although ICIs have important benefits, they also can induce adverse events, called immune-related adverse events (irAEs), which affect different tissues of the body [10]. The irAEs are related to the activation of the immune system by ICIs. These adverse events might deteriorate patients’ quality of life and may not be tolerable to some patients. When patients need to make a decision regarding the initiation of ICI treatment, they must balance these benefits with the harms associated with treatment, taking into consideration risk factors for developing these harms. Furthermore, in this decision process, it is important that patients consider their own values and preferences, including financial concerns.

The internet is a common source of health information for patients [11]. With the increased use of ICIs in cancer patients, it is important to understand the quality and content of educational websites providing information on this therapy for patients. The purpose of this study was to assess the quality and usability of available websites providing information about ICI for cancer patients.

## Materials and methods

We performed an environmental scan to systematically find and appraise the quality of currently available websites that provide patients with information regarding ICIs.

### Eligibility criteria

Considered for inclusion were websites created by US-based organizations, agencies, or companies that provided only English-language information for patients. We excluded websites providing only general information on cancer therapy or immunotherapy (not specific to immune checkpoint inhibitors), clinical practice guidelines from professional organizations, and information from medical journals, blogs, news items, and social media fora.

### Information sources

An expert librarian performed a comprehensive search in three different engines: Google, Yahoo!, and Bing from inception to 9/20/2021. For the Google search, we used the results of the top 100 from “search with omitted results”, and then we combined them with the results of “the most relevant results”. Then, we excluded the duplicates.

### Search strategy

The search terms used were: immunotherapy; "checkpoint inhibitors" OR "checkpoint inhibitor" (for Bing and Yahoo! we used the term checkpoint inhibitor); ipilimumab OR Yervoy OR pembrolizumab OR Keytruda OR nivolumab OR Opdivo OR atezolizumab OR Tecentriq OR durvalumab OR Imfinzi OR avelumab OR Bavencio OR cemiplimab OR Libtayo. To correctly perform the search, we signed off from personal accounts, cleared all history, and disabled safe search. For exporting the search engine results, we used SEOquake (www.seoquake.com), which is a free browser plugin that provides key search engine optimization metrics.

### Selection process

Two reviewers (J.I.R. and G.S.) independently screened the retrieved URLs in DistillerSR (Evidence Partners) to determine eligibility of the websites. Disagreements were resolved by consensus or with the participation of a third party (M.L.O.) if necessary.

### Quality assessment

The quality of each selected website was appraised independently by two reviewers (an internal medicine physician and a general medicine practitioner, J.I.R. and G.S., respectively). Discrepancies between the two reviewers were resolved with third party adjudication (M.L.O.).

We used previously validated standards for the development of health information resources on the internet [12, 13]. Before assessing the websites, the reviewers spent time reviewing background information about the different types of ICIs, their mechanisms of action, and the cancer(s) targeted and familiarizing themselves with the evaluation tool. The tool covers seven domains: a) accuracy of the information (i.e., information based on guidelines, textbooks, opinion, or unknown), b) completeness (i.e., discussion of irAEs [i.e., type, management, and risk factors], considerations for special populations [history of autoimmune disease, patients with organ transplant, etc.], and considerations with vaccines), c) technical elements (i.e., disclosure of funding, privacy and data protection, and ownership of website), d) design and aesthetics (i.e., visual aspects of the website, quality of visual presentation, menu [i.e., directional icons, bars, indicators, listing, indexes], typography [e.g., type size at least 12 points, use of bold type, color, different sizes], appropriate grammar, consistent layout [i.e., illustrations adjacent to the related text, paragraphs with five or fewer sentences, line lengths of 30 to 50 characters and spaces, pages that do not appear cluttered], subheadings, relevant graphics and images, appropriate type of material [i.e. text, tables, equations, audio and video], and browser compatibility), e) readability to assess if each website was written at a 6th grade level or below (calculated using the SMOG readability formula [14]), f) usability (i.e., easy navigation [e.g., well organized, index, table of contents, site map, easiness to return to previous page, frequently asked questions], internal search engine, option to download/print materials, speed, content tagged, functionality [i.e., support content]), and g) accessibility (e.g., appropriate color contrasting, easy-to-find content).

### User experience

Another reviewer (E.M.), who is a college student, assessed the user experience. We used a modified version of the US government’s recommended usability questionnaire [15]. The modified questionnaire contains 10 items with five response options (from strongly agree to strongly disagree) assessing: 1) the ease of finding the required information, 2) number of steps to reach the desired information, 3) ease of returning back after a wrong link is clicked, 4) organization of the information on the site, 5) clear labeling of the links, 6) correctness of the destination reached after clicking a link, 7) ease of learning to use the website, 8) user satisfaction with the website, 9) fulfillment of the user’s expectations, and 10) likelihood of recommending the website to others and returning back to the website in the future. We also incorporated questions previously used by Siddhanamatha et al. [16] to evaluate the perceived amount of information provided by the website about ICIs (too little, adequate, or too much), use of easily understandable words, overall rating of the website, and informativeness of the website for patients beyond information provided by health professionals.

### Synthesis methods

Each of the items in the seven domains in the quality assessment was rated with one of three responses: yes, no, or partial. Each item received an absolute score of “1” if present or “0” if absent. For each item, we used frequencies and percentages to summarize items that met quality standards. The reading levels of the included websites were assessed using means and standard deviations. For each item of the user experience we calculated the number of websites for which the user responded “strongly agree” or “somewhat agree”.

## Results

**Fig 1:**
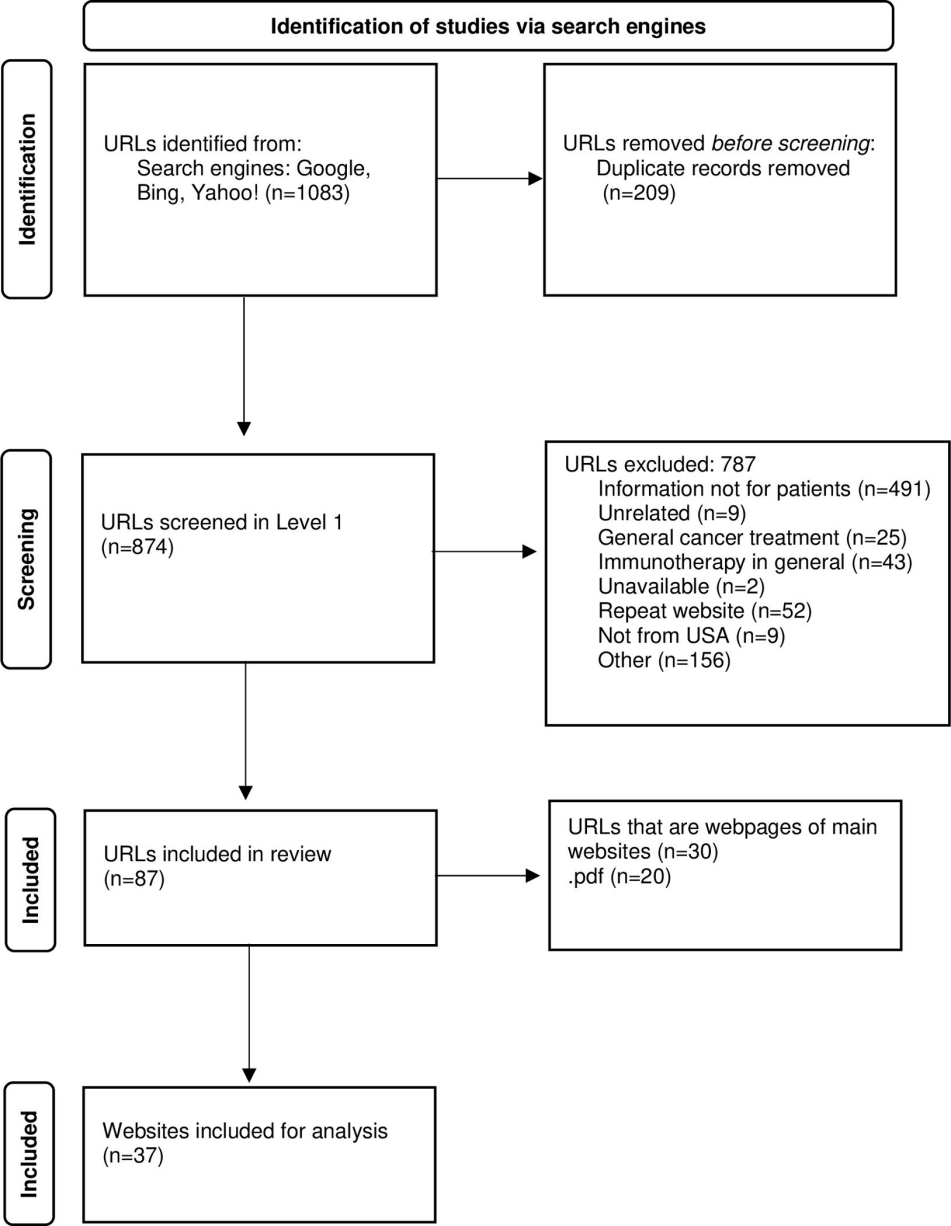
Flow diagram of immune checkpoint inhibitors website selection. *Modified from*: Page MJ, McKenzie JE, Bossuyt PM, Boutron I, Hoffmann TC, Mulrow CD, et al. The PRISMA 2020 statement: an updated guideline for reporting systematic reviews. BMJ 2021;372:n71. doi: 10.1136/bmj.n71.

Out of 1,083 URLs resulting from our search, we included 37 websites for analysis. After removal of duplicates, of the 874 URLs screened in the first step, most of them (n = 491, 56.2%) were excluded because the reviewers determined that the information provided by these websites was not directed specifically for patients. Sixty-eight (7.8%) websites were excluded because they had information about cancer treatment in general (n = 25) or about immunotherapy in general (n = 43), but not specific information about ICIs. Nine (1%) websites were excluded because they were unrelated to the topic. Fifty-two (5.9%) websites were excluded because they were duplicates. One hundred fifty-six (17.8%) websites were excluded because of format (they were YouTube videos, blog posts, social media posts, or newsletters, etc.). Nine (1%) websites were excluded because they were not created in the United States (S1 Table). The number of URLs identified in each step, the reasons for exclusions, and the final websites included are depicted in the flow diagram shown in Fig 1.

### Website characteristics

The included websites are listed in Table 1. The distribution of domain extensions was as follows: 19 were .com (57.4%), 16 were .org (43.2%), and 2 were .gov (5.4%). For the pages that provided information in PDF format, we evaluated the main website (S2 Table).

**Table 1:** General description of websites included for analysis.

*URL*	*Domain*	*Title*	*Agency/Organization*
*https://chemocare.com/chemotherapy/drug-info/default.aspx*	chemocare.com	Chemotherapy Drugs and Drugs Often Used During Chemotherapy	Chemocare
*https://medlineplus.gov/druginformation.html*	medlineplus.gov	Drugs, Herbs and Supplements	U.S. National Library of Medicine
*https://metastatictrialtalk.org/from-the-experts/immune-checkpoint-inhibitors/*	metastatictrialtalk.org	What Are Immune Checkpoint Inhibitors?	Metastatic Trial Talk
*https://moffitt.org/treatments/immunotherapy/*	moffitt.org	Immunotherapy	Moffitt Cancer Center
*https://www.asbestos.com/treatment/immunotherapy/*	asbestos.com	Mesothelioma Immunotherapy	Asbestos.com
*https://www.bavencio.com/hcp*	bavencio.com	Bavencio Avelumab	Pfizer
*https://www.breastcancer.org/treatment/immunotherapy*	breastcancer.org	Immunotherapy	Breastcancer.org
*https://www.cancer.gov/about-cancer/treatment/drugs*	cancer.gov	National Cancer Institute	National Cancer Institute
*https://www.cancer.org/cancer*	cancer.org	Cancer A-Z	American Cancer Society
*https://www.cancercenter.com/treatment-options/precision-medicine/immunotherapy*	cancercenter.com	Checkpoint Inhibitors	Cancer Treatment Centers of America
*https://www.cancerresearch.org/en-us/immunotherapy/treatment-types/immunomodulators-checkpoint-inhibitors*	cancerresearch.org	Immunomodulators	Cancer Research Foundation
*https://www.cancersupportcommunity.org/immunotherapy-cancer-it-right-you*	cancersupportcommunity.org	Immunotherapy for Cancer: Is It Right for You?	Cancer Support Community
*https://www.curemelanoma.org/patient-eng/melanoma-treatment/*	curemelanoma.org	Melanoma Treatment Options	Melanoma Research Alliance
*https://www.drugs.com/*	drugs.com	Find Drugs & Conditions	Drugs.com
*https://www.everydayhealth.com/drugs/ipilimumab*	everydayhealth.com	Ipilimumab (Yervoy)—Side Effects	Everyday Health
*https://www.foxchase.org/clinical-care/departments-programs/clinical-departments/hematology-oncology/immunotherapy*	foxchase.org	Immunotherapy Treatment	Fox Chase Cancer Center
*https://www.gene.com/patients/medicines/tecentriq*	gene.com	Tecentriq	Genentech
*https://www.imfinzi.com/*	imfinzi.com	Imfinzi (Durvalum)	AstraZeneca
*https://www.keytruda.com/*	keytruda.com	Ketruda (Pembrolizumab)	Merck
*https://www.libtayohcp.com/*	libtayohcp.com	Libtayo (Cemiplimab)	Sanofi
*https://www.lung.org/lung-health-diseases/lung-disease-lookup/lung-cancer/treatment/types-of-treatment/immunotherapy*	lung.org	Lung Cancer Immunotherapy	American Lung Association
*https://www.mayoclinic.org/drugs-supplements/ipilimumab-intravenous-route/side-effects/drg-20074841*	mayoclinic.org	Ipilimumab (Intravenous Route) Side Effects	Mayo Clinic
*https://www.mdanderson.org/patients-family/search-results.html?searchType=patient-education#_*	mdanderson.org	Patient Education	MD Anderson Cancer Center
*https://www.mdanderson.org/treatment-options/immunotherapy.html*	mdanderson.org	Immune Checkpoint Inhibitors	MD Anderson Cancer Center
*https://www.medicalnewstoday.com/articles/treating-nsclc-with-checkpoint-inhibitors*	medicalnewstoday.com	What to Know About Treating NSCLC With Checkpoint Inhibitors	Medical News Today
*https://www.medicinenet.com/*	medicinenet.com	MedicineNet	MedicineNet
*https://www.mesotheliomaguide.com/treatment/immunotherapy/*	mesotheliomaguide.com	Immunotherapy for Mesothelioma	Mesothelioma Guide
*https://www.mskcc.org/cancer-care*	mskcc.org	Cancer Care	Memorial Sloan Kettering Cancer Center
*https://www.navigatingcare.com/chemotherapy_drugs/ipilimumab-injection*	navigatingcare.com	Yervoy (Ipilimumab Injection)	Navigating Care
*https://www.nccn.org/patientresources/patient-resources/guidelines-for-patients*	nccn.org	Immunotherapy Side Effects Immune Checkpoint Inhibitors	NCCN
*https://www.opdivo.com/*	opdivo.com	Opdivo (Nivolumab)	Bristol Myers Squibb
*https://www.regionalcancercare.org/services/immunotherapy/*	regionalcancercare.org	Immunotherapy	Regional Cancer Care Associates
*https://www.rxlist.com/yervoy-drug.htm*	rxlist.com	Yervoy (Ipilimumab Injection)	RxList
*https://www.seattlecca.org/treatments/immunotherapy*	seattlecca.org	Immunotherapy	Seattle Cancer Center
*https://www.sitcancer.org/connectedold/p/patient*	patientresource.com	2020 Immunotherapy Melanoma	Society for Immunotherapy of Cancer
*https://www.tecentriq.com/*	tecentriq.com	Tecentriq Atezolizumab	Genentech
*https://www.yervoy.com/*	yervoy.com	Yervoy (Ipilimumab)	Bristol Myers Squibb

### Quality assessment

For each item assessed, the number of websites rated as “yes” is shown in Table 2. Below, we present a summary of the quality evaluation for each domain. S3 Table ranks the websites by overall score.

**Table 2:** Quality assessment by domain and item.

*Domain*	*Item*	*Yes, n (%)*
*Accuracy*	Based on guidelines or other scientific publications	24 (64.9)
	Unknown	10 (27.0)
*Completeness & Comprehensiveness*	Definition	33 (89.2)
	Management with description of each treatment	21 (56.8)
	Balance between benefits and harms of treatment	7 (18.9)
	Cost of treatment	3 (8.1)
	Monitoring	7 (18.9)
	Complications	30 (81.1)
	Areas of uncertainty	5 (13.5)
	Questions to discuss with those involved in the patient’s care	21 (56.8)
	Interaction (e.g., discussion groups, forums, etc.)	25 (67.6)
	Cases	7 (18.9)
	Subdivision of complex topics	30 (81.1)
*Completeness & Comprehensiveness specific for this study (ICI)*	Information about irAEs	28 (75.7)
	Management of irAEs of ICIs	5 (13.5)
	Explain risk factors for irAEs	15 (40.5)
	Information about vaccine contraindications, adverse events, benefits, or efficacy in patients receiving ICIs	3 (8.1)
*Technical*	Disclosure of authorship	12 (32.4)
	Disclosure of author affiliation	12 (32.4)
	Disclosure of author credentials	11 (29.7)
	Disclosure of physician credentials	11 (29.7)
	Author is recognized authority	15 (40.5)
	Disclosure of ownership	33 (89.2)
	Disclosure of sponsorship	24 (64.9)
	Editorial review process	7 (18.91)
	Clear hierarchy of evidence	3 (8.1)
	Minimal advertisement	29 (78.4)
	Statement of purpose	35 (94.6)
	General disclosures	35 (94.6)
	Identification of target audience	34 (91.9)
	Clear sources	17 (46.0)
	References	13 (35.1)
	Creative commons license	35 (94.6)
	General disclaimers	36 (97.3)
	Information on data collection and access	35 (94.6)
	Option to opt in/out of subscription service	8 (21.6)
	Message alert if cookies are used	10 (27.0)
	Message alert when leaving a secured website	1 (2.7)
	Date of content creation	3 (8.1)
	Date of last update	30 (81.1)
	Date of planned technical maintenance	1 (2.7)
	Links	11 (29.7)
	Contact information	35 (94.6)
	Feedback mechanisms	24 (64.9)
*Design*	Visual aspect of the website	37 (100)
	Quality of visual presentation	35 (94.6)
	Menu	33 (89.2)
	Typography	37 (100)
	Appropriate grammar	37 (100)
	Consistent layout	29 (78.4)
	Subheadings and chunking	36 (97.3)
	Relevant graphics and images	18 (48.7)
	Appropriate type of material, images, illustrations	19 (51.4)
	Browser compatibility	37 (100)
*Usability*	Easy navigation	36 (97.3)
	Internal search engine	32 (86.5)
	Functionality	9 (24.3)
	Registration and password protection for restricted content limited to 3 screens	0 (0.0)
	Option to download/print materials	18 (48.7)
	Large files include space for the size	18 (48.7)
	Graphic files with “mouse over” indication of graphical content	13 (35.1)
	Speed	37 (100)
	Dublin core tags	35 (94.6)
	Page does not require other computer applications for viewing	31 (81.8)
*Accessibility*	Compliance with World Wide Web Consortium 2018 guidelines	12 (32.4)
	Findability	35 (94.6)
	Appropriate color contrast	35 (94.6)
	Cultural match: appropriate language	35 (94.6)
	Cultural match: images and examples presented in realistic positive ways	35 (94.6)
*Readability*	Appropriate reading level (6th grade)	2 (5.4)
	Writing style	34 (91.9)
	Sentence construction	35 (94.6)

irAEs, immune-related adverse events; ICI, immune checkpoint inhibitors.

#### Accuracy

The information provided by 24 websites (64.9%) was based on guidelines or other scientific publications. In 10 (27%) websites, it was not possible to know the source of the information. Of the 37 websites analyzed, 7 were from pharmaceutical companies manufacturing the immune checkpoint inhibitors (opdivo.com, yervoy.com, bavencio.com, keytruda.com, imfinzi.com, libtayo.com, tecentriq.com). The content provided by the pharmaceutical companies’ websites and four other websites (cancerreaserch.org, gene.com, medicinenet.com, rxlist.com) was based on FDA information.

#### Completeness and comprehensiveness

Most of the websites (89.2%) provided a clear definition with a simple explanation of the topic ICI, and 56.8% provided information describing each treatment, how it works, mechanism of action, and contraindications. Most of the websites (81.1%) also provided information about complications. However, less than 20% of the websites had information about the balance between benefits and harms. The cost of the treatment was included in only 8.1% of the websites.

We added specific items to evaluate the completeness and comprehensiveness of the websites’ information about ICIs. We judged that 74.7% of the websites provided information about irAEs. Specifically, most of the websites listed the irAEs (cardiac, dermatological, endocrine, gastrointestinal, hematological, muscular, neurological, ocular, pulmonary, renal, systemic and rheumatic). Less than half (40.5%) of the websites explained risk factors for irAEs. Most of the websites that provided information about the risk factors denoted pre-existing autoimmune disease and patients with organ transplant as the main risk factors. Few websites (n = 5, 13.5%) included information about the management of irAEs of ICIs. Only three websites provided information about vaccine contraindications their adverse events and benefits, or efficacy in patients receiving ICIs.

#### Technical elements

Most of the websites (94.6%) provided a clear statement of purpose, declaring that the information was not meant to replace the advice of a health professional. Moreover, most of the websites gave a clear definition of the target audience (91.9%). Thirty websites (81.1%) provided the date that the website was last updated. However, only three websites (8.1%) explained clearly the hierarchy of evidence, meaning that it was not clear what information was obtained from opinions and what information was from higher levels of evidence (e.g., cohort studies, randomized controlled trials, systematic reviews). The majority of the websites (78.4%) complied with advertisement guidelines [17].

#### Design and aesthetics

All the websites had: 1) appropriate visual aspects (scroll bars and alignment), 2) acceptable typography, and 3) appropriate grammar. However, only 18 (48.7%) contained relevant graphics and images, and 19 (51.35%) used an appropriate variety of material (i.e., text, graphics, tables, equations, audio and video), appropriate cover images (i.e., to attract attention and clearly portray the purpose of the materials), and appropriate illustrations and media (i.e., no auto-play and minimal use of animation).

#### Usability

Most of the websites were judged to be easy to use. Thirty-six (97.3%) websites appeared to be well organized, had an index and a table of contents, made it easy to return to the previous page, and had a help feature and frequently asked questions. All the websites showed appropriate speed, taking less than 5 seconds for the pages to load, including text and images. Thirty-two (86.5%) websites had an internal search engine. In three of the websites, the search engine was enhanced by Google.

#### Accessibility

Under this domain, we evaluated the cultural match of the website, the appropriateness of color contrast, the findability of the content, and the availability of the website to people with disabilities. Most of the websites (94.6%) used appropriate language and images and presented examples in realistic and positive ways. We judged that 94.6% of the websites used appropriate color contrast, with font and background color contrasting, and were reader-friendly. We also determined that for most of the websites, the content was easy to find. On the other hand, we judged that only 32.4% of the websites complied with the World Wide Web Consortium 2018 guidelines, which means that the website is available to people with disabilities.

#### Readability

The mean SMOG readability score for the 37 websites was 10 (standard deviation ±2.1) with a minimum of 6 and a maximum of 16. Only 2 websites had a readability score of 6, 1 website had a score of 7, and 8 websites had a score of 8. For websites that included more than one page or several PDF documents, we calculated the average score of all pages. Most of the websites (91.9%) used a conventional writing style, with active voice and simple sentences, and 94.6% consistently gave context before presenting new information.

#### Website scores

The maximum score that a website could have if all the answers to the items were “yes” was 72. All the websites except four had a final score lower than 50.

#### User experience

In S4 Table, we present the results of the user experience. All the websites met the expectations of the person who rated the user experience, and for more than 97% of the websites, the reviewer stated that she would return to the website and would recommend it to others. However, for 27% of the websites, the reviewer perceived that the website did not always use words that could be understood. Furthermore, for 13.5% of the websites, too little information about ICIs was given.

## Discussion

To our knowledge, this is the first study to evaluate the content and quality of websites with educational material about ICI treatment. By assessing accuracy, completeness, technical elements, design and aesthetics, readability, usability, and accessibility, and navigation, we identified important differences between websites, allowing us to provide a comprehensive account of the current state of 37 patient-oriented websites on ICI treatment.

Most websites gave the complete definition, mechanism of action, and complications of ICI. More than 80% of the websites included a clear definition of the drugs and expanded on complications. However, only 20% of the websites gave information about the balance between benefits and harms. This is an important issue to address since previous studies have shown that presenting qualitative and quantitative information about benefits and risks leads to more accurate perceptions of drugs’ adverse events and benefits [18]. In a randomized controlled trial that sought to evaluate whether qualitative information, quantitative information, or a combination of the two best communicates benefits and risks, the investigators observed that providing absolute frequencies and percentages may benefit consumers by improving their drug knowledge and changing their perceptions [19]. In the present study, more than 75% of the websites provided information about irAEs. However, less than half of the websites explained risk factors for developing irAEs, and only 13.5% provided information about the management of irAEs. Only three websites gave information about vaccine contraindications, adverse events, and efficacy in patients receiving ICIs. irAEs are adverse events of ICI treatment that occur through immune activation. Almost any tissue of the body can be affected, and some patients bear a greater risk of developing them. A meta-analysis published in 2017 showed that patients with melanoma being treated with ICIs had a higher frequency of gastrointestinal and skin irAEs [20]. Patients with preexisting autoimmune diseases could have a greater risk of irAEs and flares of their autoimmune disease. In a retrospective cohort study, irAEs or autoimmune disease flares occurred in 71% of cancer patients with preexisting autoimmune disorders who were treated with ICIs, but they were easily managed [21]. Similar findings were observed in other studies, with a slightly lower incidence of flares and adverse events [22, 23]. Therefore, it is important for patients to receive clear and detailed information about risks and management of irAEs, which is largely lacking in current ICI websites.

Regarding the technical domain, most of the websites had general disclaimers, identification of the target audience, general disclosures, and contact information. More than 80% of the websites provided the date of last update. On the other hand, less than 10% of websites provided a clear hierarchy of evidence, and just 17% clearly stated the sources of the information. Almost all websites had good visual aspects, with appropriate typography and type of material. Furthermore, all the websites showed appropriate grammar. These findings indicate that the websites were strong overall in various technical aspects.

Concerningly, when we analyzed the readability of the websites, we observed that only two websites had a SMOG readability score of 6, and one website had a score of 7. The National Institutes of Health (NIH) recommend that health material be written at a grade 6–7 reading level. Our results echo those of previously published studies. In a study published in 2016, Hutchinson et al. observed that online educational material pertaining to 9 common internal medicine diagnoses used reading levels significantly above grade 6–7 [24]. Similarly, other studies have shown that the content of online educational material in other topics have on average a higher reading level than the NIH-recommended level [13, 25]. Our findings indicate that more effort should be made to provide patients with less complex information on ICI treatment that is more understandable.

We also evaluated the user experience of a user. For all the websites, the reviewer was satisfied with the experience of using the website. We also noticed that more than 25% of the websites did not always use words that were easily understood. This result is similar to that of our previously published study that evaluated the quality of websites with educational material for patients with rheumatoid arthritis [16]. In that study, more than 35% of the websites were perceived to not always use easily understandable words.

Our study has some limitations. First, we used three search engines, but there are other search engines that might find other websites. However, Google is the most popular search engine in the United States, accounting for more than 85% of all searches done in the country [26]. Another point of concern is that we excluded websites from other countries. There could be well-developed websites with important information about ICI for patients from other countries that patients from the United States can have access and get information from. However, we decided not to include other countries’ websites because the search engines we used account for IP address and would not prioritize the websites in the ranking of search results. Finally, the results of our study search would be difficult to replicate because of the complexity of the algorithms used by search engines to rank what comes up first in a search. Nevertheless, the results of our study give a picture of what is currently on the internet about ICIs for patients.

In conclusion, we have shown the strengths and weaknesses of the quality and content of current websites with information for cancer patients on ICIs, which will help organizations develop websites with detailed, high-quality information, presented in less-complex language. Patients could make better and more informed decisions if all the potential weaknesses found on the websites in our study are taken into consideration and addressed by the website’s developers. Moreover, improving the readability of websites would make them easier to understand and use, especially for people with low health literacy skills. These individuals might feel better informed with a website that follows the standards outlined, which can lead to less uncertainty about their condition and improve their self-efficacy.

## Supporting information

S1 Table Websites excluded because not from the United States. (DOCX)

S2 Table Main websites with webpages. (DOCX)

S3 Table Websites ranked by total score. (DOCX)

S4 Table User experience. (DOCX)

## References

[1] Larkin J, Chiarion-Sileni V, Gonzalez R, Grob JJ, Rutkowski P, Lao CD, et al. Five-Year Survival with Combined Nivolumab and Ipilimumab in Advanced Melanoma. N Engl J Med. 2019;381(16):1535–46. doi: 10.1056/NEJMoa1910836 31562797

[2] Dafni U, Tsourti Z, Vervita K, Peters S. Immune checkpoint inhibitors, alone or in combination with chemotherapy, as first-line treatment for advanced non-small cell lung cancer. A systematic review and network meta-analysis. Lung Cancer. 2019;134:127–40. doi: 10.1016/j.lungcan.2019.05.029 31319971

[3] Ferrara R, Imbimbo M, Malouf R, Paget-Bailly S, Calais F, Marchal C, et al. Single or combined immune checkpoint inhibitors compared to first-line platinum-based chemotherapy with or without bevacizumab for people with advanced non-small cell lung cancer. Cochrane Database Syst Rev. 2021;4(4):Cd013257. 33930176 10.1002/14651858.CD013257.pub3 PMC8092423

[4] Pasquali S, Hadjinicolaou AV, Chiarion Sileni V, Rossi CR, Mocellin S. Systemic treatments for metastatic cutaneous melanoma. Cochrane Database Syst Rev. 2018;2(2):Cd011123. doi: 10.1002/14651858.CD011123.pub2 29405038 PMC6491081

[5] Khan M, Lin J, Liao G, Tian Y, Liang Y, Li R, et al. Comparative analysis of immune checkpoint inhibitors and chemotherapy in the treatment of advanced non-small cell lung cancer: A meta-analysis of randomized controlled trials. Medicine (Baltimore). 2018;97(33):e11936. doi: 10.1097/MD.0000000000011936 30113497 PMC6113026

[6] Hargadon KM, Johnson CE, Williams CJ. Immune checkpoint blockade therapy for cancer: An overview of FDA-approved immune checkpoint inhibitors. Int Immunopharmacol. 2018;62:29–39. doi: 10.1016/j.intimp.2018.06.001 29990692

[7] Haslam A, Prasad V. Estimation of the Percentage of US Patients With Cancer Who Are Eligible for and Respond to Checkpoint Inhibitor Immunotherapy Drugs. JAMA Netw Open. 2019;2(5):e192535. doi: 10.1001/jamanetworkopen.2019.2535 31050774 PMC6503493

[8] Rizzo A, Ricci AD. Biomarkers for breast cancer immunotherapy: PD-L1, TILs, and beyond. Expert Opinion on Investigational Drugs. 2022;31(6):549–55. doi: 10.1080/13543784.2022.2008354 34793275

[9] Rihawi K, Ricci AD, Rizzo A, Brocchi S, Marasco G, Pastore LV, et al. Tumor-Associated Macrophages and Inflammatory Microenvironment in Gastric Cancer: Novel Translational Implications. Int J Mol Sci. 2021;22(8). doi: 10.3390/ijms22083805 33916915 PMC8067563

[10] Wang DY, Salem JE, Cohen JV, Chandra S, Menzer C, Ye F, et al. Fatal Toxic Effects Associated With Immune Checkpoint Inhibitors: A Systematic Review and Meta-analysis. JAMA Oncol. 2018;4(12):1721–8. doi: 10.1001/jamaoncol.2018.3923 30242316 PMC6440712

[11] McMullan M. Patients using the Internet to obtain health information: How this affects the patient–health professional relationship. Patient Education and Counseling. 2006;63(1):24–8. doi: 10.1016/j.pec.2005.10.006 16406474

[12] Abdel-Wahab N, Rai D, Siddhanamatha H, Dodeja A, Suarez-Almazor ME, Lopez-Olivo MA. A comprehensive scoping review to identify standards for the development of health information resources on the internet. PLoS One. 2019;14(6):e0218342. doi: 10.1371/journal.pone.0218342 31220126 PMC6586310

[13] Lopez-Olivo MA, des Bordes JKA, Syed MN, Alemam A, Dodeja A, Abdel-Wahab N, et al. Quality appraisal of educational websites about osteoporosis and bone health. Arch Osteoporos. 2021;16(1):28. doi: 10.1007/s11657-021-00877-x 33566216

[14] Mc Laughlin GH. SMOG grading-a new readability formula. Journal of reading. 1969;12(8):639–46.

[15] Brooke J. System Usability Scale (SUS) 1986 [Available from: http://www.usability.gov/how-to-and-tools/methods/system-usability-scale.html.

[16] Siddhanamatha HR, Heung E, Lopez-Olivo MLA, Abdel-Wahab N, Ojeda-Prias A, Willcockson I, et al. Quality assessment of websites providing educational content for patients with rheumatoid arthritis. Semin Arthritis Rheum. 2017;46(6):715–23. doi: 10.1016/j.semarthrit.2017.01.006 28258768

[17] Winker MA, Flanagin A, Chi-Lum B, White J, Andrews K, Kennett RL, et al. Guidelines for Medical and Health Information Sites on the InternetPrinciples Governing AMA Web Sites. JAMA. 2000;283(12):1600–6. 10735398 10.1001/jama.283.12.1600

[18] Schwartz LM, Woloshin S, Welch HG. Using a drug facts box to communicate drug benefits and harms: two randomized trials. Ann Intern Med. 2009;150(8):516–27. doi: 10.7326/0003-4819-150-8-200904210-00106 19221371

[19] Sullivan HW, O’Donoghue AC, Aikin KJ. Communicating Benefit and Risk Information in Direct-to-Consumer Print Advertisements: A Randomized Study. Ther Innov Regul Sci. 2015;49(4):493–502. doi: 10.1177/2168479015572370 30222437 PMC7337975

[20] Khoja L, Day D, Wei-Wu Chen T, Siu LL, Hansen AR. Tumour- and class-specific patterns of immune-related adverse events of immune checkpoint inhibitors: a systematic review. Ann Oncol. 2017;28(10):2377–85. doi: 10.1093/annonc/mdx286 28945858

[21] Tison A, Quéré G, Misery L, Funck-Brentano E, Danlos FX, Routier E, et al. Safety and Efficacy of Immune Checkpoint Inhibitors in Patients With Cancer and Preexisting Autoimmune Disease: A Nationwide, Multicenter Cohort Study. Arthritis Rheumatol. 2019;71(12):2100–11. doi: 10.1002/art.41068 31379105

[22] MenziesAM, JohnsonDB, RamanujamS, AtkinsonVG, WongANM, ParkJJ, et al. Anti-PD-1 therapy in patients with advanced melanoma and preexisting autoimmune disorders or major toxicity with ipilimumab. Ann Oncol. 2017;28(2):368–76. doi: 10.1093/annonc/mdw443 27687304

[23] Johnson DB, Sullivan RJ, Ott PA, Carlino MS, Khushalani NI, Ye F, et al. Ipilimumab Therapy in Patients With Advanced Melanoma and Preexisting Autoimmune Disorders. JAMA Oncol. 2016;2(2):234–40. doi: 10.1001/jamaoncol.2015.4368 26633184

[24] Hutchinson N, Baird GL, Garg M. Examining the Reading Level of Internet Medical Information for Common Internal Medicine Diagnoses. Am J Med. 2016;129(6):637–9. doi: 10.1016/j.amjmed.2016.01.008 26829438

[25] Kapoor K, George P, Evans MC, Miller WJ, Liu SS. Health Literacy: Readability of ACC/AHA Online Patient Education Material. Cardiology. 2017;138(1):36–40. doi: 10.1159/000475881 28571004

[26] GlobalStats S. Search engine market share United States of America. 2020.

