# Supplementary material for: Quality and content evaluation of websites with information about immune checkpoint inhibitors: An environmental scan
Source: PLoS One. 2022 Oct 10;17(10):e0275676. doi: 10.1371/journal.pone.0275676 (PMC9550065; doi:10.1371/journal.pone.0275676)
Supplement: S1 Table — (DOCX) [file pone.0275676.s001.docx]

| **S1 Table.** **Websites excluded because not from the United States** | | | |
| --- | --- | --- | --- |
| **URL** | **Title** | **Agency/Organization** | **Country** |
| https://www.cancerresearchuk.org/about-cancer/cancer-in-general/treatment/immunotherapy | Immunotherapy | Cancer Research UK | United Kingdom |
| https://www.esmo.org/content/download/124130/2352601/1/ESMO-Patient-Guide-on-Immunotherapy-Side-Effects.pdf | Patient Guide on Immunotherapy | ESMO | Switzerland |
| https://lymphoma-action.org.uk/about-lymphoma-treatment-lymphoma-targeted-drugs/checkpoint-inhibitors | Targeted treatments and antibody therapy | Lymphoma Action | United Kingdom |
| https://www.cancerresearchuk.org/about-cancer/cancer-in-general/treatment/cancer-drugs/drugs/nivolumab | Nivolumab (Opdivo) | Cancer Research UK | United Kingdom |
| https://www.cancerresearchuk.org/about-cancer/cancer-in-general/treatment/cancer-drugs/drugs/pembrolizumab | Pembrolizumab (Keytruda) | Cancer Research UK | United Kingdom |
| https://www.cancerresearchuk.org/about-cancer/cancer-in-general/treatment/cancer-drugs/drugs/ipilimumab-nivolumab | Ipilimumab and Nivolumab | Cancer Research UK | United Kingdom |
| https://www.macmillan.org.uk/cancer-information-and-support/treatments-and-drugs/ipilimumab | Ipilimumab | MacMillan Cancer Support | United Kingdom |
| https://www.cancercareontario.ca/en/drugformulary/drugs/ipilimumab | Ipilimumab | Cancer Care Ontario | Canada |
| https://www.cancerresearchuk.org/about-cancer/cancer-in-general/treatment/cancer-drugs/drugs/ipilimumab-yervoy | Ipilimumab (Yervoy) | Cancer Research UK | United Kingdom |
