# Supplementary material for: Quality and content evaluation of websites with information about immune checkpoint inhibitors: An environmental scan
Source: PLoS One. 2022 Oct 10;17(10):e0275676. doi: 10.1371/journal.pone.0275676 (PMC9550065; doi:10.1371/journal.pone.0275676)
Supplement: S2 Table — (DOCX) [file pone.0275676.s002.docx]

**S2 Table.** **Main websites with webpages**

| **Website** | **Webpages included in the website** |
| --- | --- |
| https://chemocare.com/chemotherapy/drug-info/default.aspx | https://chemocare.com/chemotherapy/drug-info/atezolizumab.aspx |
|  | https://chemocare.com/chemotherapy/drug-info/durvalumab.aspx |
|  | https://chemocare.com/chemotherapy/drug-info/ipilimumab.aspx |
|  | https://chemocare.com/chemotherapy/drug-info/Nivolumab.aspx |
|  | https://chemocare.com/chemotherapy/drug-info/Pembrolizumab.aspx |
| https://medlineplus.gov/druginformation.html | https://medlineplus.gov/druginformation.html |
|  | https://medlineplus.gov/druginfo/meds/a614048.html |
|  | https://medlineplus.gov/druginfo/meds/a614056.html |
| https://www.breastcancer.org/treatment/immunotherapy | https://www.breastcancer.org/treatment/immunotherapy |
|  | https://www.breastcancer.org/treatment/immunotherapy/keytruda |
| https://www.cancer.gov/about-cancer/treatment/drugs | https://www.cancer.gov/about-cancer/treatment/drugs/atezolizumab |
|  | https://www.cancer.gov/about-cancer/treatment/drugs/ipilimumab |
|  | https://www.cancer.gov/about-cancer/treatment/drugs/nivolumab |
|  | https://www.cancer.gov/about-cancer/treatment/types/immunotherapy |
| https://www.cancer.org/cancer | <https://www.cancer.org/cancer/breast-cancer/treatment/immunotherapy.html> |
|  | <https://www.cancer.org/cancer/esophagus-cancer/treating/immunotherapy.html> |
|  | <https://www.cancer.org/cancer/kidney-cancer/treating/immunotherapy.html> |
|  | <https://www.cancer.org/cancer/laryngeal-and-hypopharyngeal-cancer/treating/immunotherapy.html> |
|  | <https://www.cancer.org/cancer/liver-cancer/treating/immunotherapy.html> |
|  | <https://www.cancer.org/cancer/lung-cancer/treating-non-small-cell/immunotherapy.html> |
|  | https://www.cancer.org/cancer/malignant-mesothelioma/treating/immunotherapy.html |
|  | <https://www.cancer.org/cancer/melanoma-skin-cancer/treating/immunotherapy.html> |
|  | <https://www.cancer.org/cancer/oral-cavity-and-oropharyngeal-cancer/treating/immunotherapy.html> |
|  | https://www.cancer.org/treatment/treatments-and-side-effects/treatment-types/immunotherapy.html |
|  | https://www.cancer.org/treatment/treatments-and-side-effects/treatment-types/immunotherapy/what-is-immunotherapy.html |
| https://www.curemelanoma.org/patient-eng/melanoma-treatment/ | https://www.curemelanoma.org/patient-eng/melanoma-treatment/combination-therapy-for-melanoma/nivolumab-opdivo-ipilimumab-yervoy/ |
|  | https://www.curemelanoma.org/patient-eng/melanoma-treatment/immunotherapy/ |
|  | https://www.curemelanoma.org/patient-eng/melanoma-treatment/immunotherapy/ipilimumab-yervoy/ |
| https://www.drugs.com/ | <https://www.drugs.com/keytruda.html> |
|  | <https://www.drugs.com/opdivo.html> |
|  | https://www.drugs.com/sfx/ipilimumab-side-effects.html |
|  | <https://www.drugs.com/tecentriq.html> |
| https://www.medicinenet.com/ | https://www.medicinenet.com/ipilimumab_yervoy/article.htm |
|  | <https://www.medicinenet.com/side_effects_of_ipilimumab_yervoy/side-effects.htm> |
| https://www.mskcc.org/cancer-care | <https://www.mskcc.org/cancer-care/diagnosis-treatment/cancer-treatments/immunotherapy> |
|  | https://www.mskcc.org/cancer-care/patient-education/nivolumab-01 |
|  | https://www.mskcc.org/cancer-care/types/bladder/treatment/immunotherapy-bladder |
|  | https://www.mskcc.org/cancer-care/types/melanoma/treatment/immunotherapy-melanoma |
| https://www.nccn.org/patientresources/patient-resources/guidelines-for-patients | <https://www.nccn.org/patients/guidelines/content/PDF/immunotherapy-se-ici-patient.pdf> |
| https://www.sitcancer.org/connectedold/p/patient | <https://www.patientresource.com/userfiles/file/ImmunoMelanoma2020.pdf> |
| https://www.mdanderson.org/patients-family/search-results.html?searchType=patient-education#_ | https://www.mdanderson.org/patient-education/General-Internal-Medicine/Immune-Checkpoint-Inhibitor-Side-Effects.pdf |
|  | https://www.mdanderson.org/patient-education/General-Internal-Medicine/Immunotherapy-Side-Effect-Myocarditis.pdf |
|  | <https://www.mdanderson.org/patient-education/General-Internal-Medicine/Immunotherapy-Side-Effect-Low-Blood-Counts-(Cytopenia).pdf> |
|  | https://www.mdanderson.org/patient-education/General-Internal-Medicine/Immunotherapy-Side-Effect-Colitis.pdf |
|  | https://www.mdanderson.org/patient-education/General-Internal-Medicine/Immunotherapy-Side-Effect-Nephritis.pdf |
|  | https://www.mdanderson.org/patient-education/General-Internal-Medicine/Immunotherapy-Side-Effect-Myositis-Myalgia.pdf |
|  | https://www.mdanderson.org/patient-education/General-Internal-Medicine/Immunotherapy-Side-Effect-SICCA-Syndrome.pdf |
|  | https://www.mdanderson.org/patient-education/General-Internal-Medicine/Immunotherapy-Side-Effect-Arthritis-Arthralgia.pdf |
|  | https://www.mdanderson.org/patient-education/General-Internal-Medicine/Immunotherapy-Side-Effect-Pancreatitis.pdf |
|  | https://www.mdanderson.org/patient-education/General-Internal-Medicine/Immunotherapy-Side-Effect-Pneumonitis.pdf |
|  | https://www.mdanderson.org/patient-education/General-Internal-Medicine/Immunotherapy-Side-Effect-Hypophysitis.pdf |
|  | https://www.mdanderson.org/patient-education/General-Internal-Medicine/Immunotherapy-Side-Effect-Type-1-Diabetes.pdf |
|  | https://www.mdanderson.org/patient-education/General-Internal-Medicine/Immunotherapy-Side-Effect-Thyroiditis.pdf |
|  | https://www.mdanderson.org/patient-education/General-Internal-Medicine/Immunotherapy-Side-Effect-Skin-Inflammation.pdf |
|  | https://www.mdanderson.org/patient-education/General-Internal-Medicine/Immunotherapy-Side-Effect-Liver-Inflammation.pdf |
|  | https://www.mdanderson.org/patient-education/General-Internal-Medicine/Immunotherapy-Side-Effect-Myasthenia-Gravis.pdf |
|  | <https://www.mdanderson.org/patient-education/General-Internal-Medicine/Immunotherapy-Side-Effect-Meningitis-and-Encephalitis.pdf> |
|  | https://www.mdanderson.org/patient-education/General-Internal-Medicine/Immunotherapy-Side-Effect-Peripheral-Neuropathy.pdf |
|  | https://www.mdanderson.org/patient-education/General-Internal-Medicine/Immunotherapy-Side-Effect-Transverse-Myelitis.pdf |
|  | https://www.mdanderson.org/patient-education/General-Internal-Medicine/Immunotherapy-Side-Effect-Guillain-Barre-Syndrome.pdf |
