# Supplementary material for: Quality and content evaluation of websites with information about immune checkpoint inhibitors: An environmental scan
Source: PLoS One. 2022 Oct 10;17(10):e0275676. doi: 10.1371/journal.pone.0275676 (PMC9550065; doi:10.1371/journal.pone.0275676)
Supplement: S3 Table — (DOCX) [file pone.0275676.s003.docx]

**S3 Table.** **Websites ranked by total score.**

| **Website** | **Score** |
| --- | --- |
| https://www.regionalcancercare.org/services/immunotherapy/ | 25 |
| https://www.seattlecca.org/treatments/immunotherapy | 25 |
| https://metastatictrialtalk.org/from-the-experts/immune-checkpoint-inhibitors/ | 28 |
| https://www.foxchase.org/clinical-care/departments-programs/clinical-departments/hematology-oncology/immunotherapy | 31 |
| https://www.cancercenter.com/treatment-options/precision-medicine/immunotherapy | 34 |
| https://www.rxlist.com | 34 |
| https://www.cancersupportcommunity.org/immunotherapy-cancer-it-right-you | 35 |
| https://www.curemelanoma.org/patient-eng/melanoma-treatment/ | 35 |
| https://www.mdanderson.org/patients-family/search-results.html?searchType=patient-education#_ | 35 |
| https://moffitt.org/treatments/immunotherapy/ | 36 |
| https://www.mskcc.org/cancer-care | 36 |
| https://www.everydayhealth.com/drugs/ipilimumab | 37 |
| https://www.navigatingcare.com/chemotherapy_drugs/ipilimumab-injection | 39 |
| https://www.tecentriq.com/ | 39 |
| https://www.mdanderson.org/treatment-options/immunotherapy.html | 40 |
| https://chemocare.com/chemotherapy/drug-info/default.aspx | 41 |
| https://medlineplus.gov/druginformation.html | 41 |
| https://www.breastcancer.org/treatment/immunotherapy | 41 |
| https://www.medicalnewstoday.com/articles/treating-nsclc-with-checkpoint-inhibitors | 41 |
| https://www.gene.com/patients/medicines/tecentriq | 42 |
| https://www.bavencio.com/hcp | 43 |
| https://www.lung.org/lung-health-diseases/lung-disease-lookup/lung-cancer/treatment/types-of-treatment/immunotherapy | 43 |
| https://www.cancer.gov/about-cancer/treatment/drugs | 44 |
| https://www.drugs.com/ | 44 |
| https://www.imfinzi.com/ | 44 |
| https://www.mayoclinic.org/drugs-supplements/ipilimumab-intravenous-route/side-effects/drg-20074841 | 44 |
| https://www.medicinenet.com/ | 44 |
| https://www.libtayohcp.com/ | 45 |
| https://www.cancer.org/cancer | 47 |
| https://www.yervoy.com/ | 47 |
| https://www.opdivo.com/ | 48 |
| https://www.asbestos.com/treatment/immunotherapy/ | 49 |
| https://www.mesotheliomaguide.com/treatment/immunotherapy/ | 49 |
| https://www.keytruda.com/ | 50 |
| https://www.sitcancer.org/connectedold/p/patient | 51 |
| https://www.cancerresearch.org/en-us/immunotherapy/treatment-types/immunomodulators-checkpoint-inhibitors | 52 |
| https://www.nccn.org/patientresources/patient-resources/guidelines-for-patients | 56 |
