# Supplementary material for: Quality and content evaluation of websites with information about immune checkpoint inhibitors: An environmental scan
Source: PLoS One. 2022 Oct 10;17(10):e0275676. doi: 10.1371/journal.pone.0275676 (PMC9550065; doi:10.1371/journal.pone.0275676)
Supplement: S4 Table — (DOCX) [file pone.0275676.s004.docx]

**S4 Table.** **User experience.** Number of websites (n=37) for which the user responded “strongly agree” or “somewhat agree”

| *Item* | *No. (%)* |
| --- | --- |
| It was easy to find the information I wanted | 28 (75.7) |
| The number of steps it took to get me where I wanted to go was acceptable | 35 (94.6) |
| If I clicked the wrong link, I knew how to go back | 36 (97.3) |
| The information on this site was well organized | 27 (73.0) |
| The links were labeled clearly | 29 (78.4) |
| When I clicked on a link, I ended up where I expected | 35 (94.6) |
| It was easy to learn how to use the website | 32 (86.5) |
| I am satisfied with my experience using this website | 37 (100) |
| The website meets my expectations | 35 (94.6) |
| I would recommend this website to others | 36 (97.3) |
| I would return to this website | 36 (97.3) |
| ***Added questions*** |  |
| Amount of information in the website |  |
| *Just right* | 27 (73.0) |
| *Too little information* | 5 (13.5) |
| *Too much information* | 5 (13.5) |
| The website always uses the words that you understand |  |
| *Yes* | 27 (73.0) |
| *No* | 10 (27.0) |
| Overall rating of the website |  |
| *Excellent* | 16 (43.2) |
| *Good* | 14 (37.8) |
| *Fair* | 7 (18.9) |
| *Poor* | 0 (0) |
